# House calls by community health workers and public health nurses to improve adherence to isoniazid monotherapy for latent tuberculosis infection: a retrospective study

**DOI:** 10.1186/1471-2458-13-894

**Published:** 2013-09-28

**Authors:** Alicia H Chang, Andrea Polesky, Gulshan Bhatia

**Affiliations:** 1 Department of Medicine, Division of Infectious Diseases and Geographic Medicine, Stanford University, 300 Pasteur Drive, Grant Building S-101, MC-5107, Stanford, CA 94305, USA; 2 Santa Clara County Tuberculosis Clinic, Santa Clara Valley Health and Hospital System, 976 Lenzen Ave, San Jose, CA 95126, USA; 3 Department of Medicine, Division of Mycobacterial Diseases and International Health, Santa Clara Valley Medical Center, 751 S. Bascom Ave, San Jose, CA 95128, USA

**Keywords:** Latent tuberculosis infection, LTBI, Adherence, Isoniazid, Community health workers, Public health nurses, House calls, Hepatitis, Rifampin

## Abstract

**Background:**

Patient adherence to isoniazid (INH) monotherapy for latent tuberculosis infection (LTBI) has been suboptimal despite its proven efficacy. Various strategies have been studied to improve adherence, but all have been based at a clinic or treatment program. At the Santa Clara Valley Tuberculosis Clinic, it was our practice to refer a subset of high-risk LTBI patients to the Public Health Department for monthly follow-up at home instead of at the clinic. Our goal was to assess whether house calls by community health workers and public health nurses affected INH adherence or frequency of adverse effects.

**Methods:**

We retrospectively studied 3918 LTBI patients who received INH. At the discretion of the treating physician, 986 (25.2%) received house calls instead of clinic follow-up. Home-based follow-up included language translation, medication delivery, assessment of compliance with pill counts, monitoring for adverse effects, and active tracking of noncompliant patients. We assessed differences in patient characteristics, treatment completion, and reasons for treatment discontinuation between patients followed at home versus in the clinic. Multivariate analyses to address possible referral bias or confounding were performed using logistic regression.

**Results:**

More patients followed with house calls completed INH treatment (90% home versus 73.2% clinic). This was the case across all subgroups of patients, including those with historically the lowest adherence: patients from correctional and rehabilitation facilities (77.8% home versus 46.9% clinic), postpartum women (86.4% home versus 55.6% clinic), and patients aged between 18 and 35 years (87% home versus 63.1% clinic). After adjusting for age, place of birth, referral category (TB contacts/skin test converters, correctional/rehabilitation patients, postpartum women, tuberculin positive patients from other screening), and prescribed INH regimen duration (9 versus 6 months), home-based follow-up of LTBI patients was a significant predictor of treatment completion (AOR 2.94, 95% CI: 2.33, 3.71). Patients followed at home were 21% more likely to complete therapy (ARR 1.21, p<0.001). Risk of adverse effects was similar between the two types of follow-up.

**Conclusion:**

Home-based follow-up of LTBI patients taking isoniazid was associated with improved treatment completion and no increase in adverse effects regardless of patient characteristics or prescribed duration of INH therapy.

## Background

Targeted screening for latent tuberculosis infection (LTBI) followed by treatment with isoniazid (INH) is an important facet of tuberculosis (TB) control programs in the US [1]. Studies have shown that LTBI treatment with INH is 69% to 93% effective in preventing the development of active TB, but effectiveness depends greatly on the level of adherence [2-4]. The U.S. Centers for Disease Control and Prevention set the target completion rate in high-risk individuals at 85% [5]. In most clinic settings, however, completion of LTBI treatment falls significantly short of this goal and ranges from 47% to 60% [6,7]. Various strategies to improve LTBI treatment adherence have been evaluated for different high-risk groups but none has proven effective across the board [6,8-10]. In Santa Clara County, California, many of the LTBI patients are recent immigrants or refugees who have significant social obstacles to receiving and completing INH treatment. In an effort to improve adherence for patients at high risk of treatment failure, the Santa Clara County Tuberculosis Clinic used a strategy that involved public health nurses assessing a subset of LTBI patients in their homes every month for the duration of treatment. To assess the efficacy of this approach, we conducted a retrospective evaluation of the effect of house calls on INH treatment completion and incidence of adverse effects.

## Methods

### Study site and patients

The Santa Clara County Tuberculosis Clinic is a publicly funded TB referral center. The study included all LTBI patients with positive tuberculin skin tests (TST+) and normal chest radiographs who began INH treatment from January 1, 2000 to December 31, 2002. Patients were referred from community physicians; immigration services; correctional and rehabilitation facilities; school, work, antenatal, and homeless shelter screening; and Public Health Department contact investigations. Patients who discontinued INH due to unavoidable reasons (pregnancy, index case with INH-resistant TB, death from unrelated causes, patient relocation) were excluded. The Investigational Review Board at the Santa Clara Valley Medical Center granted a waiver for the study and gave ethical approval for publication of the results.

### INH treatment and follow-up

INH treatment consisted of 300-milligram (mg) daily tablets in adults and doses of 10 to 15 mg/kilogram/day in children. During the study period, there was an overlap of both 6 and 9 months of INH prescriptions as new recommendations from the Centers for Disease Control and Prevention took effect [1]. At the discretion of the treating physician, patients who were identified at increased risk for not completing INH treatment, and in particular high TB-risk patients (children younger than six, contacts to TB cases, and TST converters) were assigned to monthly home follow-up instead of monthly clinic visits. Both community health workers, who assisted with medication delivery, translation, and assessment of compliance, and public health nurses, who monitored adverse effects and conducted active tracking of noncompliant patients, performed house calls. Patients experiencing side effects returned to the clinic for evaluation while continuing home-based follow-up.

Compliance was assessed by clinic attendance (for those with clinic follow-up), pill count, and number of INH refills received. Patients who failed three consecutive appointments were considered lost to follow-up. Completion of therapy was defined by receiving at least 6 months of INH within 9 months of time. We chose this definition because taking at least 6 months of INH is 69% protective against active TB [2].

For patients taking other medications with known hepatotoxicity or with co-morbidities such as chronic viral hepatitis or alcohol abuse, baseline and monthly liver function tests (LFTs) were ordered at the discretion of the physician. INH treatment was stopped if significant adverse reactions developed or if patients became pregnant. Individual charts were reviewed for patients who stopped treatment due to abnormal LFTs. The definition of definite INH-related hepatitis was an alanine aminotransferase three times the upper limit of normal with accompanying symptoms or five times the upper limit of normal regardless of symptoms [11].

### Analysis

Main outcome measures were proportions of patients completing INH treatment and of patients developing adverse effects among those followed at home versus at the clinic. Differences in proportions were analyzed with the Chi-square and Fisher’s exact tests. Differences in means and distributions were compared using the t-test or the Wilcoxon rank-sum test as appropriate. Multivariate analyses were conducted using logistic regression. Variables that approached statistical significance in univariate analyses (p<0.20) were tested in multivariate models. Adjusted relative risks (RR) were calculated from the adjusted odds ratios using the procedure described by Zhang and Yu [12]. Confidence intervals for the RRs were estimated using the substitution method [13]. Risk of adverse reactions and INH-hepatitis were analyzed using multivariate logistic regression. Analyses were performed using SAS statistical software (version 9.3, SAS Institute, Cary, North Carolina). Multiple comparisons were addressed using the Hochberg adjustment [14].

## Results

### Patient characteristics

A total of 3918 TST+ patients with normal chest radiographs were prescribed INH; 25.2% were assigned to have home-based follow-up while the remaining 74.8% received solely clinic-based follow-up (Table 1). Most patients were foreign-born (87.2%). The three most frequent countries of origin were Mexico (50.4%), Vietnam (10.2%), and the Philippines (8.2%). There were more children under six years of age in the house call group, as well as more patients who were TST converters and TB case contacts. In contrast, very few patients from correctional and rehabilitation facilities were assigned to have house calls (0.9%). The need for translation services was similar for all patients regardless of which type of follow-up they received.

**Table 1:** Characteristics of patients with latent tuberculosis infection

**Characteristic**		**Home follow-up**^**a **^**(N=986)**		**Clinic follow-up**^**b **^**(N=2932)**		**p-value**^**c**^
		**N**	**(%)**	**N**	**(%)**	
Female		549	(55.7)	1467	(50.0)	0.002
Median age		22		24		<0.001
(1^st^ and 3^rd^ quartile)		(10–31)		(13–35)		
Age categories:						<0.001
	< 6 years old	141	(14.3)	140	(4.8)	
	6 to < 18	277	(28.1)	967	(33.0)	
	18 to < 35	399	(40.5)	1085	(37.0)	
	35 and older	169	(17.1)	740	(25.2)	
Place of birth:						0.008
	Latin America	592	(60.0)	1573	(53.7)	
	Asia	243	(24.7)	837	(28.6)	
	USA	75	(7.6)	268	(9.1)	
	Africa	22	(2.2)	48	(1.6)	
	Europe	20	(2.0)	82	(2.8)	
	Unknown	34	(3.5)	124	(4.2)	
Needed translation service^d^		60	(6.1)	176	(6.0)	0.93
Patient referral reason:						
	TST converters and TB case contacts^e^	220	(22.3)	229	(7.8)	<0.001
	Correction and rehabilitation^f^	9	(0.9)	226	(7.7)	
	Postpartum women^g^	59	(6.0)	169	(5.8)	
	TST+^h^ from screening	698	(70.8)	2308	(78.7)	
Prescribed 9H^i^ regimen		555	(56.3)	1595	(54.4)	0.30

^a^Patients assigned home-based follow-up with a community health worker and public health nurse.

^b^Patients with clinic-based follow-up.

^c^Significance testing with chi-square test or Wilcoxon rank-sum test.

^d^Patients who spoke languages other than Spanish, Tagalog, Vietnamese, Cantonese, or Mandarin, and required use of a phone translation service or a family member for communication.

^e^Recent tuberculin skin test converters and household contacts to active tuberculosis cases.

^f^Patients from correctional and drug and alcohol rehabilitation facilities.

^g^Women treated more than 2 months after delivery of child.

^h^TST+: Tuberculin skin test positive from routine screening.

^i^ Nine months of isoniazid as opposed to six months of isoniazid.

### INH treatment completion

The proportion of patients completing at least 6 months of INH ranged from 46.9% to 95.7% depending on patient category (Table 2). The highest non-adherence was seen in patients from correctional and rehabilitation facilities and in postpartum women. The age group 18 to 35 had the highest noncompliance. With home follow-up, completion improved across all subgroups of patients. In the multivariate analysis, the final explanatory variables of treatment completion were type of follow-up (home vs. clinic), duration of INH regimen (9 months vs. 6 months), age (<6, 6 to <18, 18 to <35, and 35 and older), patient referral reason (TB contact/TST converter, correctional patient, postpartum patient, and all other TST reactors), and region of birth. After adjusting for these variables, patients followed at home by public health nurses and community health workers had a three-fold increase in the odds of treatment completion compared to those followed in the clinic. This adjusted odds ratio corresponded to a 21% increase in the probability of completing INH treatment for patients with home visits (Table 3). To better understand why patients failed to complete at least six months of INH, we examined the reasons for INH discontinuation according to the type of follow-up assigned (Table 4) and found that those assigned to house calls were much less likely to refuse to continue treatment (7.6% versus 23.2% clinic follow-up, p <0.001).

**Table 2:** Latent tuberculosis patients completing INH treatment, stratified by type of follow-up

	**Home follow-up**^**a **^**N=986**		**Clinic follow-up**^**b **^**N=2932**			
**Patient characteristic**	**Completed**^**c**^	**(%)**^**d**^	**Completed**	**(%)**	**p-value**^**e**^	**Risk ratio**^**f**^
All patients	887	(90.0)	2147	(73.2)	<0.001^g^	1.23
Female	500	(91.1)	1068	(72.8)	0.99	1.25
Age categories:					<0.001	
Age<6	135	(95.7)	129	(92.1)		1.04
Age 6 to <18	258	(93.1)	813	(84.1)		1.11
Age 18 to <35	347	(87.0)	685	(63.1)		1.38
Age 35 and older	147	(87.0)	520	(70.3)		1.24
Place of birth:					<0.001	
Latin America	526	(88.9)	1124	(71.5)		1.24
Asia	227	(93.4)	652	(77.9)		1.20
USA	66	(88.0)	185	(69.0)		1.28
Africa	21	(95.5)	34	(70.8)		1.35
Europe	19	(95.0)	66	(80.5)		1.18
Unknown	28	(82.4)	86	(69.4)		1.19
Patient referral reason:					<0.001	
Correctional/rehabilitation^h^	7	(77.8)	106	(46.9)		1.66
TST converters/TB contacts^i^	199	(90.5)	177	(77.3)		1.17
Postpartum women	51	(86.4)	94	(55.6)		1.55
TST+^j^ from screening	630	(90.3)	1770	(76.9)		1.17
Prescribed 9 months isoniazid^k^	508	(91.5)	1226	(76.9)	<0.001	1.19

^a^Patients assigned home-based follow-up with community health workers and public health nurses.

^b^Patients with only clinic-based follow-up.

^c^Completed at least 6 months of isoniazid.

^d^Percentage of number completed over total number in category, listed in Table 1.

^e^Cochran-Mantel-Haenszel test except as noted, all p-values adjusted for multiple comparisons.

^f^Unadjusted “risk” ratio of completing treatment of home versus clinic follow-up for each patient category.

^g^Chi-square test.

^h^Patients from correctional and drug and alcohol rehabilitation facilities.

^i^Recent tuberculin skin test converters and household contacts to active tuberculosis cases.

^j^TST+: Tuberculin skin test positive.

^k^Prescribed 9 months instead of 6 months of isoniazid.

**Table 3:** Factors associated with completion of at least 6 months of isoniazid treatment

**Risk factor**	**OR**^**a**^	**95% CL**		**AOR**^**b**^	**95% CL**		**ARR**^**c**^	**95% CL**^**d**^	
Home follow-up^e^	3.27	2.62	4.09	2.94	2.33	3.71	1.21	1.18	1.24
9 months of isoniazid	1.50	1.29	1.74	0.88	0.73	1.05	0.97	0.92	1.01
Female	1.04	0.89	1.21						
Age categories:									
Age<6	6.80	4.11	11.25	5.40	3.19	9.13	1.07	1.06	1.08
Age 6 to <18	2.71	2.23	3.30	2.64	2.10	3.32	1.11	1.09	1.12
Age 18 to <35	ref			ref			ref		
Age 35 and older	1.21	1.00	1.45	1.18	0.96	1.44	1.05	0.99	1.10
Patient referral reason:									
Correctional/rehabilitation	0.23	0.18	0.31	0.43	0.32	0.57	0.59	0.47	0.71
TST converters/TB contacts^f^	1.30	0.99	1.70	1.23	0.93	1.63	1.04	0.98	1.10
Postpartum women	0.44	0.33	0.59	0.64	0.47	0.87	0.80	0.67	0.94
TST+^g^ from screening	ref			ref			ref		
Place of birth:									
Africa	1.34	0.72	2.50	1.29	0.67	2.49	1.07	0.87	1.21
Asia	1.60	1.21	2.13	1.62	1.18	2.22	1.09	1.03	1.14
Europe	1.83	1.03	3.25	1.93	1.06	3.53	1.10	1.01	1.16
Latin America	1.17	0.91	1.52	1.25	0.93	1.67	1.06	0.98	1.13
Unknown	0.95	0.62	1.45	1.06	0.67	1.68	1.02	0.87	1.14
USA	ref			ref			ref		

^a^Analysis by logistic regression.

^b^Adjusted OR. Multivariate model included: type of follow-up, regimen, age (categories), patient referral reason, and place of birth.

^c^Adjusted Risk Ratio, calculated from adjusted OR.

^d^Adjusted Risk Ratio 95% confidence limits, calculated from adjusted OR.

^e^Home-based follow-up with public health nurses and community health workers.

^f^Tuberculin skin test converters or contacts of cases with active tuberculosis.

^g^TST+: Tuberculin skin test positive.

**Table 4:** Reasons for not completing INH treatment for latent tuberculosis infection

**Reason**	**Home follow-up**^**a**^		**Clinic follow-up**^**b**^		**P-value**^**c**^
	**N=986**	**(%)**	**N=2932**	**(%)**	
Adverse reaction	24	(2.4)	105	(3.6)	0.24
Abnormal liver enzymes^d^	3	(0.3)	40	(1.4)	0.02
*INH –related hepatitis*^*e*^	*1*	*(0.1)*	*23*	*(0.8)*	*0.06*^*f*^
Rash	7	(0.7)	30	(1.0)	0.58
Other symptoms^g^	14	(1.4)	35	(1.2)	0.58
Refused/Lost^h^	75	(7.6)	680	(23.2)	<0.001

^a^Home-based follow-up with community health workers and public health nurses.

^b^Clinic-based follow-up.

^c^Analysis by chi-square or Fisher’s exact test, adjusted for multiple comparisons.

^d^Any abnormality of liver enzymes associated with treatment discontinuation.

^e^Subset of patients with abnormal liver enzymes with isoniazid-related hepatitis, based on chart review.

^f^p-value for INH-related hepatitis only.

^g^Any other intolerable symptom that necessitated treatment discontinuation such as headache, dizziness, fatigue, among others.

^h^Refused to continue treatment or were lost to follow-up.

### Adverse reactions leading to INH discontinuation

There were few adverse reactions requiring treatment discontinuation. These included abnormal LFTs, abdominal pain, nausea, rash, headache, dizziness, and fatigue (Table 4). The occurrence of adverse events was 3.3% for the entire cohort and similar between the two types of follow-up. We adjusted for age (<35 years vs. ≥35 years), and postpartum status (yes vs. no). Age 35 was chosen as the cut-off to reflect the historical age threshold used to determine whether INH should be offered to TST+ patients who were at low risk for TB reactivation [15]. There was no association between adverse events and type of patient follow-up after adjusting for age and referral reason (AOR 0.72, 95% CI 0.46-1.14, p=0.16).

Abnormal LFTs were infrequent, but more common in clinic follow-up patients (1.4% vs 0.3% house calls, p=0.02). Many of these patients had abnormal LFTs prior to starting INH. Development of definite INH-related hepatitis (not due to pre-existing viral hepatitis, for example) was rare, and occurred in only 1 (0.1%) patient with home follow-up and 23 (0.8%) patients with clinic follow-up (p=0.06). The incidence of hepatitis due to INH was 0.4% in patients younger than 35 and 1.3% in those 35 and older. Women aged 18 to 35 who were treated with INH after childbirth appeared to have similar incidence of hepatitis to other age-matched women (1.9% vs. 1.0%, p=0.29). After adjusting for age and postpartum state, there was a trend towards decreased INH-related hepatitis in the patients with home follow-up (AOR 0.15, 95% CI: 0.02-1.08, p=0.06).

## Discussion

INH monotherapy remains the mainstay of LTBI treatment, and adherence will continue to be a significant concern due to the long duration of therapy. Four months of rifampin is an alternate regimen for LTBI that is of shorter duration, has higher patient adherence than 9 months of INH [16,17], and is likely more cost-effective [18]. However, efficacy studies of rifampin versus INH are limited [19]. Recently, a 3-month regimen of INH and rifapentine once weekly with DOT had very high patient adherence and similar efficacy as 6 or 9 months of INH [20,21] in both HIV positive and negative patients. However, this regimen is not recommended for young children, HIV patients on antiretroviral therapy, or pregnant women [22]. The medications alone are also 16 times more expensive than INH and although the regimen may be cost-effective or cost-saving at the societal level [23], it could be difficult in resource-limited settings.

Our study demonstrates that house calls may be an effective method to increase INH treatment adherence to the U.S. national target of 85%. Several strategies to improve treatment adherence have been examined in the past. These have included the use of cultural case-management, education, peer support, financial incentives, direct observed therapy (DOT), and active tracking of patients at high risk of failing to complete treatment [6,24]. Some of the strategies reported, such as coupling DOT with methadone treatment [1], are very specific for the intended patient cohort and are less useful in other populations. Non-physician health workers such as pharmacists [9] or case-manager nurses [25,26] have also been found effective in following LTBI patients. However all of the interventions studied to date have been clinic-based. House calls to LTBI patients constitute a method that could be applied across many types of patient populations.

One of the limitations of the study was the non-randomized method of assigning patients to receive either house calls or clinic follow-up. The assignment reflected current practice in our clinic and relied on the experience and discretion of the physician and nurse practitioner. It is clear from the different distribution of patient characteristics (Table 1) that clinicians considered the patient’s age, risk of failing to complete treatment, and risk of progression to active tuberculosis when choosing the type of follow-up, and this may have introduced bias or confounding into the study. For example, it is possible that TB contacts are more likely to complete treatment because of patients’ perception of risk, and these patients were also more likely to be assigned home follow-up, particularly because the index case could also be receiving DOT at home. Arguing against this was the finding that TB contacts and routine TST+ patients had similar completion rates within each type of follow-up (Table 2). For example, both TB contacts and TST+ patients from screening had 77% completion with clinic follow-up versus 90% with house calls. Instead, awareness of insufficient resources and staffing might have biased physicians into assigning fewer high-risk patients for home follow-up than they might have otherwise. It was also our practice to refer high-risk patients who missed three clinic appointments to have home follow-up, thereby biasing the home follow-up group with patients more likely to fail treatment completion. Therefore, it is also possible that the true effect of house calls on INH adherence was attenuated. Because patient characteristics between the groups were different, we made an effort to address possible confounders with multivariate analysis. We recognize that residual confounding may remain. For example, there were more patients with abnormal liver enzymes in the clinic follow-up group. This could be explained by a tendency to keep patients with comorbidities in the clinic or a lower threshold for clinic physicians to elicit symptoms and test for liver abnormalities that might otherwise have gone undetected. We acknowledge these limitations of our study. A randomized clinical trial would be helpful in validating home follow-up as a way to increase INH treatment adherence, but might never be funded.

When considering the impact of house calls to follow LTBI patients, the added expense of this approach needs to be measured. The true cost of home-based follow-up in our county is difficult to quantify because both community health workers and public health nurses had other clinical responsibilities and we do not know the exact fraction of time that these health workers specifically spent on house calls. An assessment of cost would need to take into account the time saved by the staff normally involved in the care of LTBI patients in the standard clinic-based follow-up. Several other parameters also need to be evaluated. For example, similar to other TB clinics, we require our patients to return monthly to the clinic for symptom review and assessment of compliance [27] unless they are followed at home. Although these monthly clinic visits are effective in reducing the incidence of severe INH-related hepatitis [28], they may be too burdensome on the patient and negatively affect INH adherence. It is possible that only one component of the house calls, such as medication delivery via health workers, is sufficient. Until the shorter, alternate treatment regimens for LTBI become more affordable and widely available, the strategy presented here of home-based follow-up of patients on INH, and the associated cost variables, merit further exploration and study.

## Conclusions

House calls to follow LTBI patients taking INH were associated with improved treatment completion regardless of patient age, referral reason, place of birth, or prescribed length of INH therapy. Monthly home-based follow-up with language-appropriate assessments, delivery of medications, and active tracking of noncompliant patients could be an effective method of increasing adherence to INH monotherapy for LTBI.

## Abbreviations

TB: Tuberculosis; LTBI: Latent tuberculosis infection; INH: Isoniazid; TST: Tuberculin skin test positive; TST: Tuberculin skin test; LFTs: Liver function tests; DOT: Direct observed therapy.

## Competing interests

The authors declare that they have no competing interests.

## Authors’ contributions

AHC performed the analyses and drafted the manuscript. AP conceived of the study. AP and GB helped draft the manuscript. All authors read and approved the final manuscript.

## Authors’ information

AHC is Instructor of Medicine, in the division of Infectious Diseases and Geographic Medicine at the Stanford University School of Medicine. AP is the Director of the Tuberculosis Clinic in Santa Clara County. GB, former director of the Tuberculosis Clinic in Santa Clara County, is currently a staff physician at the clinic.

## Pre-publication history

The pre-publication history for this paper can be accessed here:

http://www.biomedcentral.com/1471-2458/13/894/prepub
